# Successful treatment of acute myocardial injury of Duchenne muscular dystrophy with steroids: a case report

**DOI:** 10.1186/s13019-023-02159-8

**Published:** 2023-03-03

**Authors:** Merve Oğuz, Dolunay Gürses, Furkan Ufuk, Münevver Yılmaz, Olcay Güngör

**Affiliations:** 1 Department of Pediatric Cardiology, University of Pamukkale, Kinikli, 20100 Denizli, Turkey; 2 Department of Radiology, University of Pamukkale, Kinikli, 20100 Denizli, Turkey; 3 Department of Pediatric Neurology, University of Pamukkale, Kinikli, 20100 Denizli, Turkey

**Keywords:** Duchenne muscular dystrophy, Acute chest pain, Cardiac magnetic resonance imaging, Corticosteroid

## Abstract

**Background:**

Duchenne muscular dystrophy (DMD) is an X-linked muscular disease which is caused by the absence of dystrophin. Troponin elevation with acute chest pain may indicate acute myocardial injury in these patients. We report a case of DMD that presented with ACP and troponin elevation, who was diagnosed with acute myocardial injury, and successfully treated with corticosteroids.

**Case presentation:**

A 9-year-old with DMD was admitted to the emergency department with the complaint of acute chest pain. His electrocardiogram (ECG) revealed inferior ST elevation and serum troponin T was elevated. The transthoracic echocardiography (TTE) demonstrated inferolateral and anterolateral hypokinesia with depressed left ventricular function. An ECG-gated coronary computed tomography angiography ruled out acute coronary syndrome. Cardiac magnetic resonance imaging revealed mid-wall to sub-epicardial late gadolinium enhancement at the basal to the mid inferior lateral wall of the left ventricle and corresponding hyperintensity on T2-weighted imaging, consistent with acute myocarditis. A diagnosis of acute myocardial injury associated with DMD was made. He was treated with anticongestive therapy and 2 mg/kg/day of oral methylprednisolone. Chest pain resolved the next day, and ST-segment elevation returned to normal on the third day. Troponin T decreased in the sixth hour of oral methylprednisolone treatment. TTE on the fifth day revealed improved left ventricular function.

**Conclusion:**

Despite advances in contemporary cardiopulmonary therapies, cardiomyopathy remains the leading cause of death in patients with DMD. Acute chest pain attacks with elevated troponin in patients with DMD without coronary artery disease may indicate acute myocardial injury. Recognition and appropriate treatment of acute myocardial injury episodes in DMD patients may delay the development of cardiomyopathy.

## Introduction

Duchenne muscular dystrophy (DMD) is an X-linked inherited myopathy that causes progressive skeletal and cardiac muscle disease. DMD's critical features are chronic cardiac muscle inflammation and subsequent fibrotic tissue deposition. Heart lesions were described in the earliest DMD reports, and cardiomyopathy is now the leading cause of death. Early diagnosis and appropriate treatment of cardiomyopathy in DMD patients are vital in improving overall outcomes. Acute chest pain attacks with elevated troponin in patients with DMD without coronary artery disease may indicate acute myocardial injury, a sign of progression to cardiomyopathy.

## Case presentation

A 9-year-old male with Duchenne muscular dystrophy (DMD) was admitted to the emergency department with the complaint of acute chest pain (ACP) that had been present for the last six hours. He denied any viral prodrome, palpitations, shortness of breath. His medical history was significant for receiving a diagnosis of DMD at five years of age, due to the deletion of exons 22–28 of the DMD gene. After diagnosis, deflazacort therapy started at a dose of 18 mg per day and was continuously taken. Left ventricular (LV) functions (LV ejection fraction of 62%) and wall motions were within normal limits on an echocardiogram obtained 12 months before the admission to the emergency department.

**Fig. 1:**
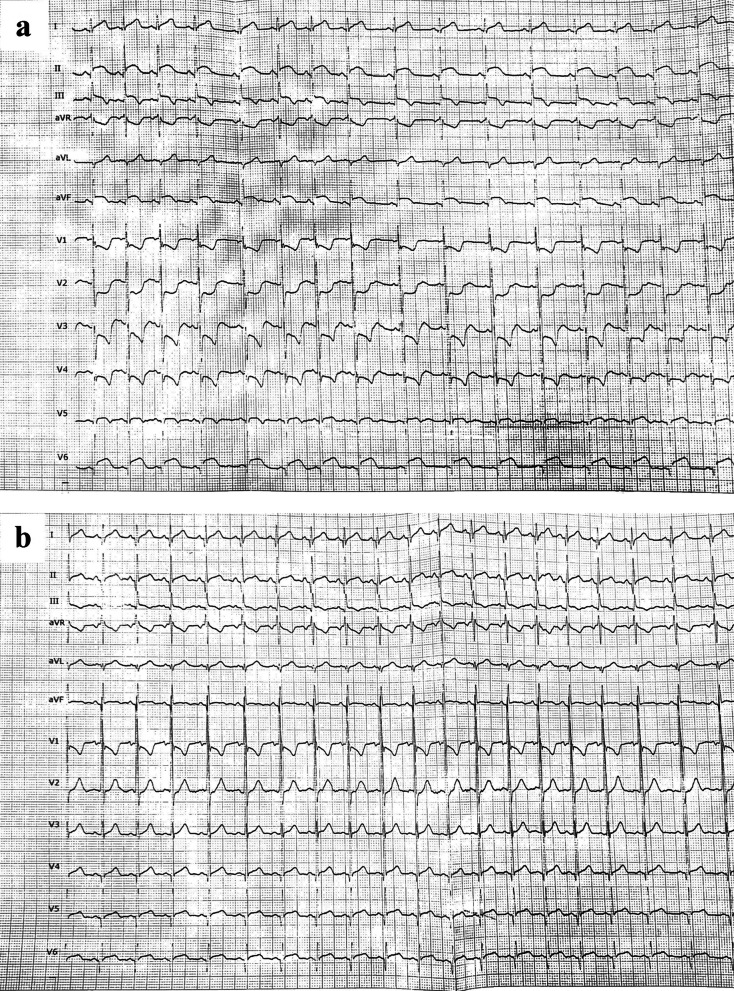
**a, b** Electrocardiogram on admission to the emergency department reveals convex ST elevation in leads II, III, aVF, and V6, with reciprocal ST depression in V1-3 leads (**a**). Electrocardiogram on the third day of treatment reveals regression of ST segment elevation (**b**)

**Fig. 2:**
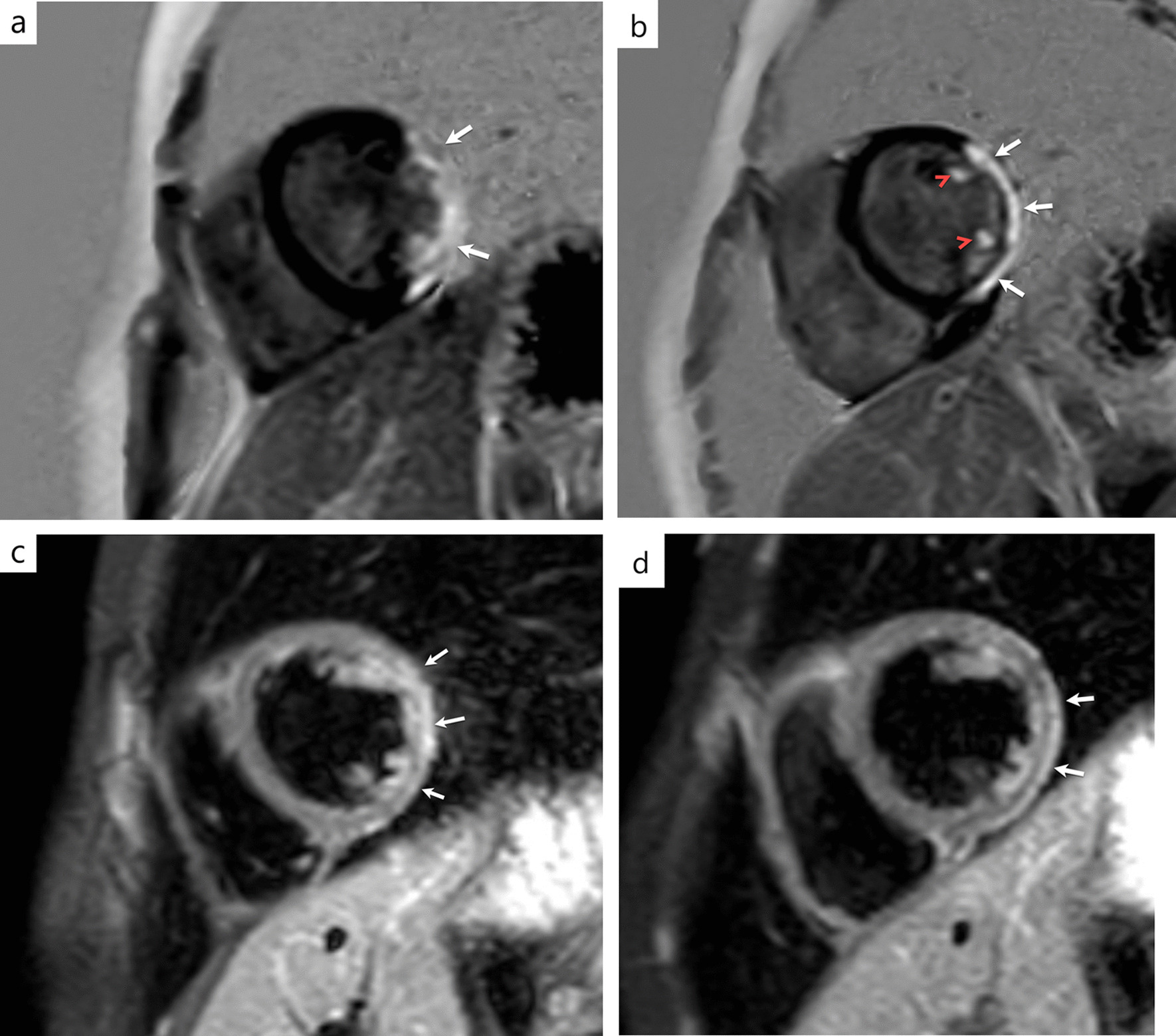
**a–d** Short-axis late gadolinium-enhanced cardiac magnetic resonance (MR) images with phase-sensitive inversion recovery (PSIR) demonstrate mid-wall to subepicardial late gadolinium enhancement at the basal to the mid inferior lateral wall (**a** and **b**, arrows) with papillary muscle enhancement (**b**, arrowheads), corresponding hyperintensity on T2-weighted imaging (**c** and **d**, arrows)

The physical examination at the current admission revealed he was afebrile, blood pressure was 126/84 mmHg, heart rate was 90 bpm, and respiratory rate was 26 breaths/min. Cardiac examination revealed a new-onset cardiac murmur and a gallop rhythm. The initial 12-lead electrocardiogram revealed convex ST elevation in leads II, III, aVF, and V6, with reciprocal ST depression in V1-3 leads (Fig. 1a). Laboratory analysis revealed an elevated serum troponin T (3.24 ug/L; normal range, 0.0–0.014 ug/L), serum CK-MB (300 ug/L; normal range, 0–5 ug/L), and brain natriuretic peptide (1860 ng/L; normal range, 0–125 ug/L) levels. Acute phase reactants, including C-reactive protein, were within normal limits. Molecular testing for myocarditis-associated viruses was negative for all viruses. The Troponin T values obtained in additional samples collected at the 6th and 12th hours afterward were also markedly elevated (i.e., 4,5 ug/L and 7,1 ug/L, respectively). The chest radiography was unremarkable and transthoracic echocardiography (TTE) demonstrated inferolateral and anterolateral hypokinesia with depressed LV function (ejection fraction of 46%) and mild mitral regurgitation. An ECG-gated coronary computed tomography angiography (CTA) was performed with the suspicion of acute myocardial infarction. Coronary CTA ruled out acute coronary syndrome. Cardiac MRI (Magnetic Rezonance Imaging) was obtained on the same day. Cardiac MRI revealed mid-wall to sub-epicardial late gadolinium enhancement at the basal to the mid inferior lateral wall of the left ventricle and corresponding hyperintensity on T2-weighted imaging, consistent with acute myocarditis. Moreover, LV papillary muscles were also affected (Fig. 2).

**Fig. 3:**
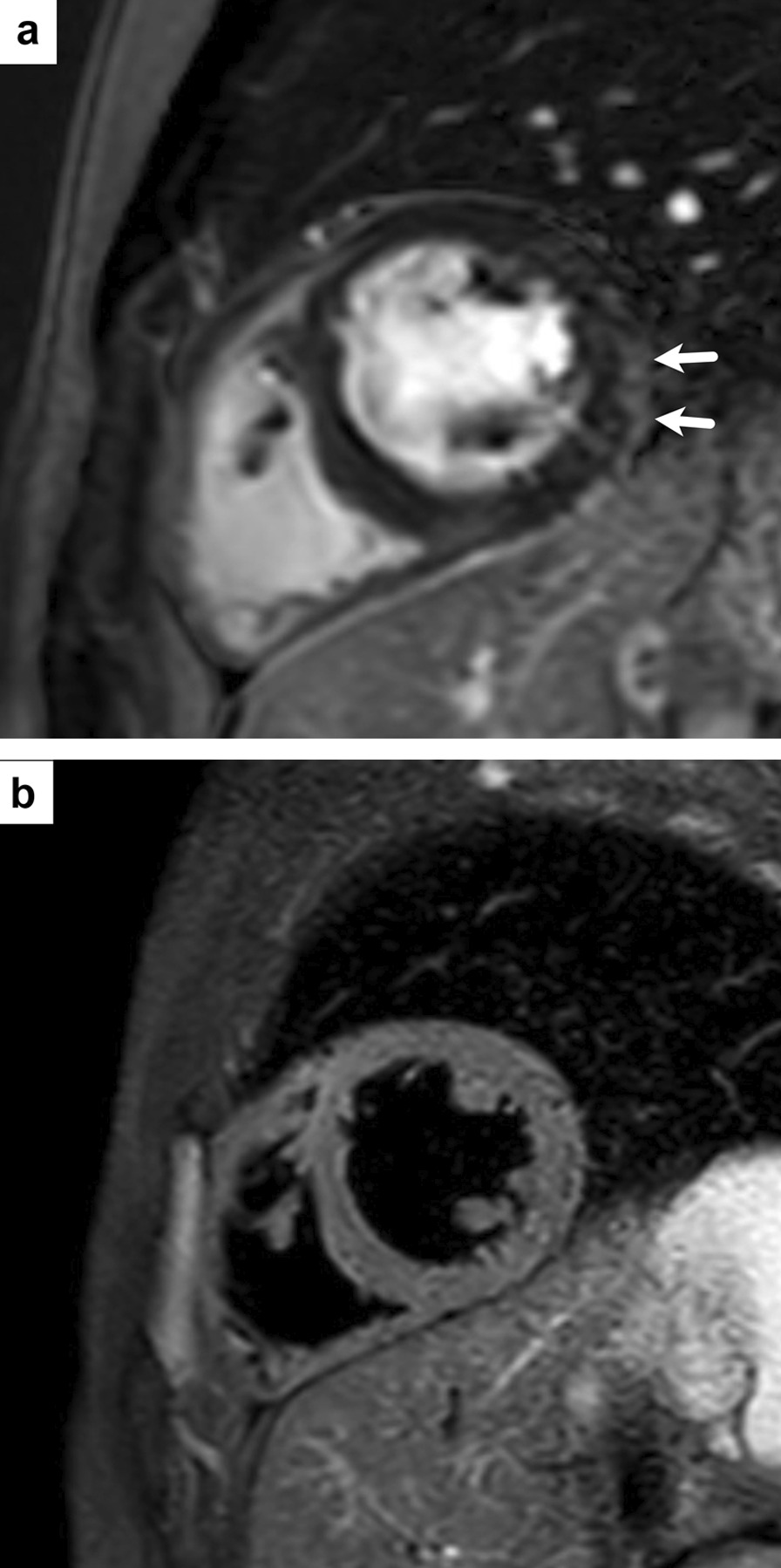
Cardiac MR images obtained four months later revealed that the findings had regressed. **a** Short-axis late gadolinium-enhanced cardiac magnetic resonance (MR) image with phase-sensitive inversion recovery (PSIR) demonstrate minimal residual subepicardial late gadolinium enhancement at the basal to the mid inferior lateral wall (arrows), and **b** no myocardial edema on T2-weighted image

A diagnosis of acute myocardial injury associated with DMD was made. He was treated with anticongestive therapy (including enalapril, spironolactone and carvedilol). Additionally, deflazacort treatment was stopped, and he was treated with 2 mg/kg/day of oral methylprednisolone. Chest pain resolved the next day, and ST-segment elevation returned to normal on the third day (Fig. 1b). Troponin T decreased in the sixth hour of oral methylprednisolone treatment, and on the fifth day, troponin T was 0.18 ng/ml. TTE on the fifth day revealed improved LV function (ejection fraction of 54%) and the patient was discharged on the sixth day of hospitalization. Cardiac MRI obtained in the fourth month after discharge showed significant improvement in the pathological findings in the LV walls (Fig. 3). LV ejection fraction was 56% with TTE at the first year follow-up.

## Discussion

Duchenne muscular dystrophy (DMD) is a rare disease that affects 1 in 3500–7000 male births worldwide and is characterized by inflammation and progressive degeneration of the skeletal and cardiac muscles resulting from mutations in the dystrophin gene [1]. Approximately 40% of deaths are due to cardiac causes. Cardiomyopathy manifests with gradual-onset systolic dysfunction and arrhythmias [2]. Because DMD patients have limited physical activity, they do not present symptoms until severe ventricular dysfunction occurs. Unfortunately, traditional biomarkers used in the assessment of DMD-associated heart failure have limited utility. Troponin is released in the context of DMD cardiac disease, likely secondary to the loss of membrane integrity and elevated troponin levels are observed in DMD and may vary with disease progression [3, 4].

In a small number of case reports, ACP, troponin elevation, and acute LV dysfunction have been reported in DMD patients [5–9]. In a report of two DMD patients with laboratory evidence of acute myocardial cell injury, it was emphasized that DMD cardiomyopathy may be associated with episodes of acute cell injury rather than gradual progressive loss of cardiac functions. The authors suggested that these episodes may be followed by regeneration that could delay the development of cardiac dysfunction [5]. In a series of eight DMD patients presenting with ACP, Hor et al. [10] demonstrated low LV ejection fraction during episodes and progressive fibrosis assessed by cardiac MRI, indicating that these episodes may be significant in the progression to primary cardiomyopathy. It is unclear whether these attacks occur spontaneously or are triggered by viral infection, physiological stress, or comorbid diseases. However, it is thought that cardiac myocytes with dystrophin deficiency may accumulate these recurrent episodic insults with age. We diagnosed our patient presenting with ACP as having acute myocardial injury associated with DMD based on his troponin elevation regressed with treatment, convex ST elevation on ECG follow-up, cardiac MRI findings at presentation and regression at follow-up, and absence of viral prodrome or detectable infectious agent and negative acute phase reactants. Previous studies have suggested that cardiomyopathy associated with neuromuscular diseases involving the dystrophin-glycoprotein complex may be due to ischemic damage, and positron emission tomography has demonstrated reduced coronary reserves in asymptomatic DMD patients with normal ventricular size and function and normal coronary vessels [11, 12]. Although the acute severe onset in our case seemed consistent with acute coronary syndrome, our patient’s coronary vessels were normal according to the CTA imaging.

The cardiac effects of corticosteroid therapy in DMD patients have not been well characterized. However, corticosteroid use has been associated with benefits such as improved overall cardiac function and delayed onset of cardiomyopathy [13]. In the literature, only one DMD patient with ACP, troponin elevation, and acute LV dysfunction was given oral pulse corticosteroid therapy [6]. In another report, fa DMD patient who could not obtain the deflazacort they were prescribed and used low-dose prednisolone instead, was presented with ACP, troponin elevation, acute LV dysfunction, and these symptoms regressed when deflazacort treatment was restarted [13].

Although we gave supportive treatments to our patient, the regression of chest pain, troponin elevation and ECG changes after the initiation of pulse steroid therapy suggests that steroid therapy was successful in this acute myocardial injury episode. In addition, the improvement in ventricular function with treatment supports the view expressed by Ramaciotti et al. [5] that regeneration of the cardiac myocytes may be observed in DMD patients.

Prospective studies with larger series are needed to prove the efficacy of steroids in the treatment of these acute myocardial injury episodes with chest pain, troponin elevation, and ECG changes in DMD patients.

However, studies including more extensive series are needed on this subject.

## Conclusion

Acute myocardial injury should be considered in DMD patients presenting with ACP. Recognition and appropriate treatment of acute myocardial injury episodes in DMD patients may delay the development of cardiomyopathy.

## Data Availability

Written informed consent was obtained from the patient for the publication of this case report and any accompanying images.
